# Testicular Metastasis From Small Cell Lung Carcinoma: A Case Report

**DOI:** 10.7759/cureus.100048

**Published:** 2025-12-25

**Authors:** Rim Alami, Reyzane El Mjabber, Asmaa Naim, Nabil Ismaili, Sanaa El Majjaoui

**Affiliations:** 1 Radiation Oncology, Cheikh Khalifa International University Hospital, Casablanca, MAR; 2 Radiation Oncology, Mohammed VI University of Health Sciences (UM6SS), Casablanca, MAR; 3 Radiotherapy, Cheikh Khalifa International University Hospital, Casablanca, MAR; 4 Radiotherapy, Mohammed VI University of Health Sciences (UM6SS), Casablanca, MAR; 5 Medical Oncology, Mohammed VI University of Health Sciences (UM6SS), Casablanca, MAR

**Keywords:** chemotherapy, rare case, sanctuary site, small-cell lung carcinoma, testicular metastasis

## Abstract

Testicular involvement from small cell lung carcinoma (SCLC) is extremely uncommon and diagnostically challenging. Awareness of this rare metastatic pattern is essential, particularly in patients presenting with new scrotal symptoms during disease progression.

We report the case of a 61-year-old North African male patient with previously treated SCLC who developed progressive right testicular enlargement at metastatic relapse. Scrotal ultrasound described a 41-mm heterogeneous intratesticular mass. Serum alpha fetoprotein and β-HCG were within normal limits. No orchiectomy was performed due to disseminated disease, declining performance status, and the lack of therapeutic benefit of local surgery. Ultrasound images could not be retrieved, but the radiology report was available. The patient underwent systemic chemotherapy rechallenge and palliative radiotherapy.

This case illustrates the diagnostic challenges of testicular metastasis in advanced SCLC, highlights the sanctuary-site concept, and emphasizes the importance of clinical context when histologic confirmation is not feasible.

## Introduction

Small cell lung carcinoma (SCLC) accounts for 10-15% of lung cancers and is characterized by aggressive behavior, rapid growth, and early dissemination [1]. Common metastatic sites include lymph nodes, liver, bone, brain, and adrenal glands. Testicular metastases from solid tumors are rare, observed in approximately 0.04% of autopsies [2], most frequently originating from prostate, lung, gastrointestinal tract, melanoma, and kidney malignancies.

Metastatic involvement of the testis by SCLC is exceptionally uncommon. Several cases have been reported in the literature [3-11], including early descriptions by Stein et al. [3] and Rosser & Gerrard [8]. Testicular masses in adults typically suggest primary germ-cell tumors or lymphoma; however, metastasis should be considered in elderly patients or cases with known disseminated malignancy.

We present a rare case of presumed right testicular metastasis in a patient with SCLC, emphasizing the diagnostic challenges when histological confirmation cannot be obtained and illustrating the decision-making process in advanced disease.

## Case presentation

A 61-year-old North African man, with no significant comorbidities, was diagnosed with limited-stage SCLC of the lung. Initial treatment consisted of six cycles of etoposide-cisplatin (EP) and thoracic radiotherapy (60 Gy in 30 fractions), followed by prophylactic cranial irradiation (25 Gy in 10 fractions). Imaging showed partial response and subsequent disease stability according to RECIST v1.1 criteria.

Approximately six months later, he developed bone metastases and received second-line chemotherapy with paclitaxel-carboplatin for eight cycles, achieving disease control for nine months.

At relapse, he presented with headaches, diffuse bone pain, right adrenal pain, and new right testicular swelling. He denied prior genitourinary symptoms. Physical examination revealed a firm, non-transilluminating enlarged right testicle. The contralateral testis and epididymis were normal.

Serum alpha fetoprotein (AFP) and β-HCG levels were obtained and found to be within normal limits. This biochemical profile made a primary germ-cell tumor less likely and supported the suspicion of secondary testicular involvement in the context of disseminated SCLC.

No orchiectomy was performed because the patient’s disease was widely metastatic, his performance status was declining, and histologic confirmation was unlikely to change management. In this context, invasive procedures such as orchiectomy were considered clinically inappropriate, as local surgical control of the testicular lesion would not have provided therapeutic benefit nor altered the overall prognosis.

Ultrasound images could not be retrieved due to the absence of archival imaging files in our institution's PACS for this examination. However, the ultrasound report was available and described an enlarged right testis that had been replaced by a solid heterogeneous intratesticular mass measuring 41 mm, with irregular margins and altered echotexture. No microlithiasis, calcifications, or cystic components were mentioned. Color Doppler evaluation was reported as showing increased vascularity.

**Figure 1:**
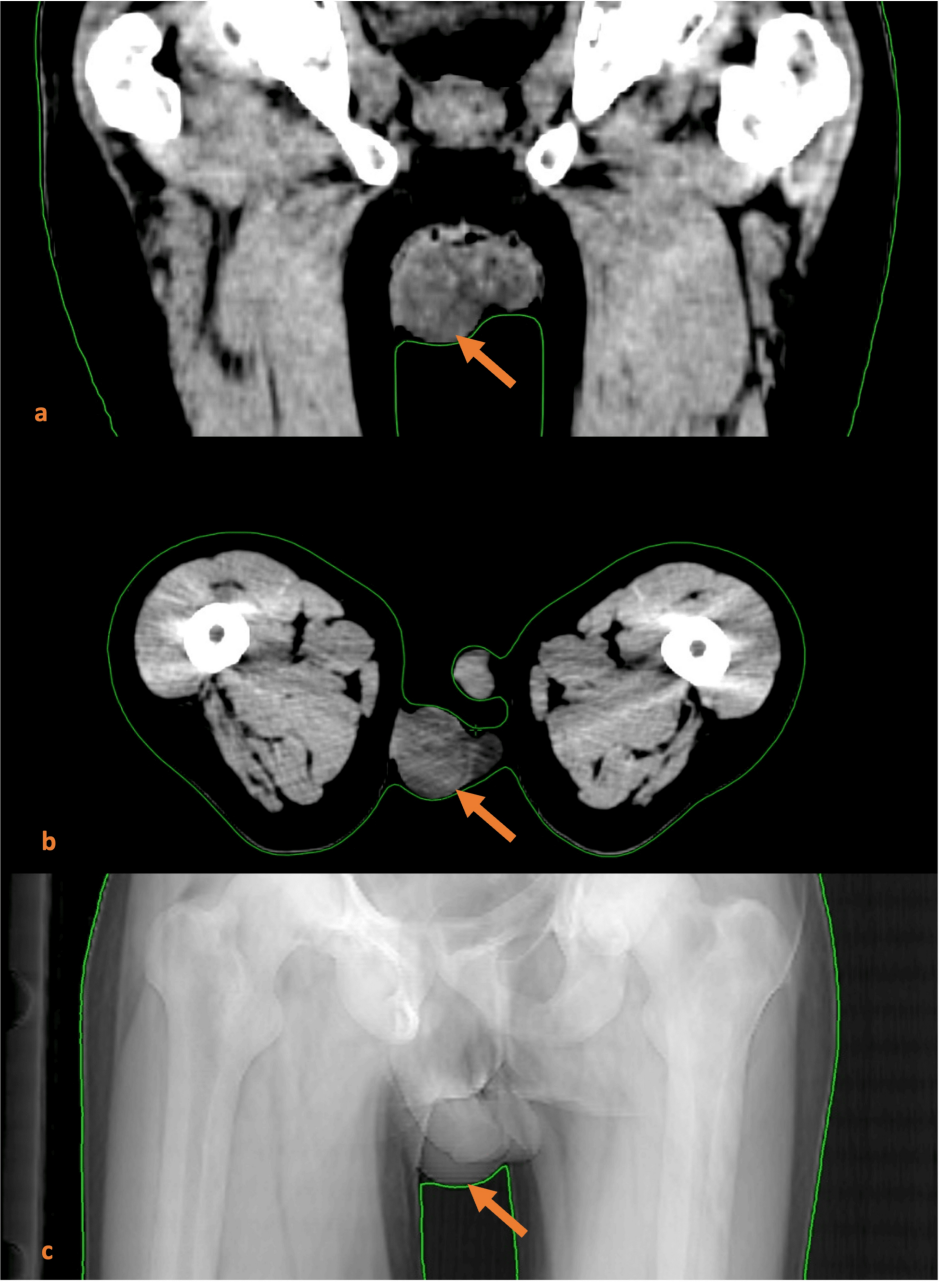
Dosimetric CT scan demonstrating the 41-mm solid heterogeneous testicular mass (orange arrow) (a) Coronal CT treatment planning slice showing a well-defined soft-tissue lesion centered within the right testis. (b) Axial CT scan confirming the intratesticular location of the heterogeneous mass, without evidence of adjacent tissue invasion. (c) Pelvic radiograph showing a right scrotal soft-tissue opacity corresponding to the underlying testicular lesion.

A thoraco-abdomino-pelvic CT scan revealed stable pulmonary lesions and progressive adrenal and bone metastases. Brain MRI showed nodular osseous involvement. The mass was also visible on the dosimetric CT performed for radiotherapy planning (Figure 1).

Given that relapse occurred more than six months after completion of first-line EP chemotherapy, the disease was classified as a sensitive relapse according to international guidelines. A rechallenge with the EP protocol was therefore initiated. The regimen consisted of etoposide 100 mg/m² administered intravenously on Days 1-3, combined with cisplatin 75 mg/m² intravenously on Day 1, repeated every 21 days. The patient completed four cycles without dose reductions or delays.

Concurrently, palliative radiotherapy was delivered to symptomatic metastatic sites. Whole-brain radiotherapy was administered with a total dose of 20 Gy in 5 fractions, given once daily over one week, for analgesic and anti-edema purposes in the context of skull and dural metastatic involvement. Additionally, adrenal radiotherapy was performed with a total dose of 30 Gy in 10 fractions, targeting the right adrenal lesion responsible for persistent pain, with a planning target volume of 5mm.

The patient tolerated systemic therapy and radiotherapy well, with no significant acute toxicity reported. Hematologic tolerance was acceptable, without grade ≥3 cytopenias. Radiotherapy was completed without interruption, and no neurological or gastrointestinal adverse effects were observed.

Follow-up CT imaging after three months showed radiological stabilization of thoracic, adrenal, and bone lesions. Brain MRI demonstrated no new lesions and resolution of previously described inflammatory changes.

To provide a clearer overview of the sequence of clinical events and therapeutic interventions, a detailed timeline of the patient’s disease course is presented in Table 1.

**Table 1:** Chronological timeline of the patient’s disease course and management This table summarizes the sequence of clinical events, imaging findings, diagnostic decisions, and therapeutic interventions from the initial diagnosis of limited-stage SCLC to the development of presumed testicular metastasis and subsequent management. The timeline highlights disease progression, treatment responses, and key decision points including the rationale for avoiding invasive diagnostic procedures. SCLC: Small cell lung carcinoma

Time Point	Clinical Event
Initial diagnosis (T0)	Limited-stage SCLC diagnosed. First-line chemotherapy with etoposide–cisplatin (6 cycles). Thoracic radiotherapy: 60 Gy in 30 fractions. Prophylactic cranial irradiation: 25 Gy in 10 fractions.
Post-treatment follow-up	Radiologic disease stability according to RECIST v1.1.
First progression	Development of bone metastases. Second-line chemotherapy: paclitaxel + carboplatin (8 cycles). Good tolerance and prolonged disease control.
+ 9 months	New progression with headaches, bone pain, and onset of right testicular swelling.
Imaging findings	Scrotal ultrasound: 41-mm heterogeneous intratesticular mass (right testis). CT scan: adrenal and bone progression, stable thoracic lesions. Brain MRI: osseous involvement.
Diagnostic decisions	No orchiectomy (widespread disease, declining performance status, minimal clinical benefit). Tumor markers not obtained.
Treatment	EP rechallenge (sensitive relapse > 6 months). Palliative radiotherapy to brain and adrenal metastases.
3-month evaluation	Radiologic stabilization on CT. Brain MRI unremarkable.

## Discussion

Testicular metastases from solid tumors are rare, and involvement by SCLC is exceptional. Although uncommon, more than a dozen cases of SCLC testicular metastasis have now been reported, underscoring that this presentation, while rare, is an established phenomenon in advanced disease. Only a limited number of case reports provide sufficient clinical details for meaningful comparison with our case. The age at presentation is generally older than that for primary testicular cancers, with a mean age of around 57 years [2].

In this case, the diagnosis of testicular metastasis was presumptive, based on the known history of metastatic SCLC, rapid enlargement of the right testis, ultrasound findings consistent with malignant infiltration, absence of features suggestive of benign lesions and absence of contralateral abnormalities.

Orchiectomy or biopsy remains the gold standard for confirming metastatic disease [1,6]. However, in patients with widespread progression, declining performance status, or situations where histology would not alter management, invasive diagnostic procedures may be omitted. Similar situations have been described in other reports where clinical context guided diagnosis when histologic confirmation was not feasible [7,10].

In this patient, AFP and β-HCG levels were normal, a finding that is characteristic of metastatic testicular involvement from SCLC rather than primary germ-cell tumors, which typically produce elevated markers.

**Figure 2:**
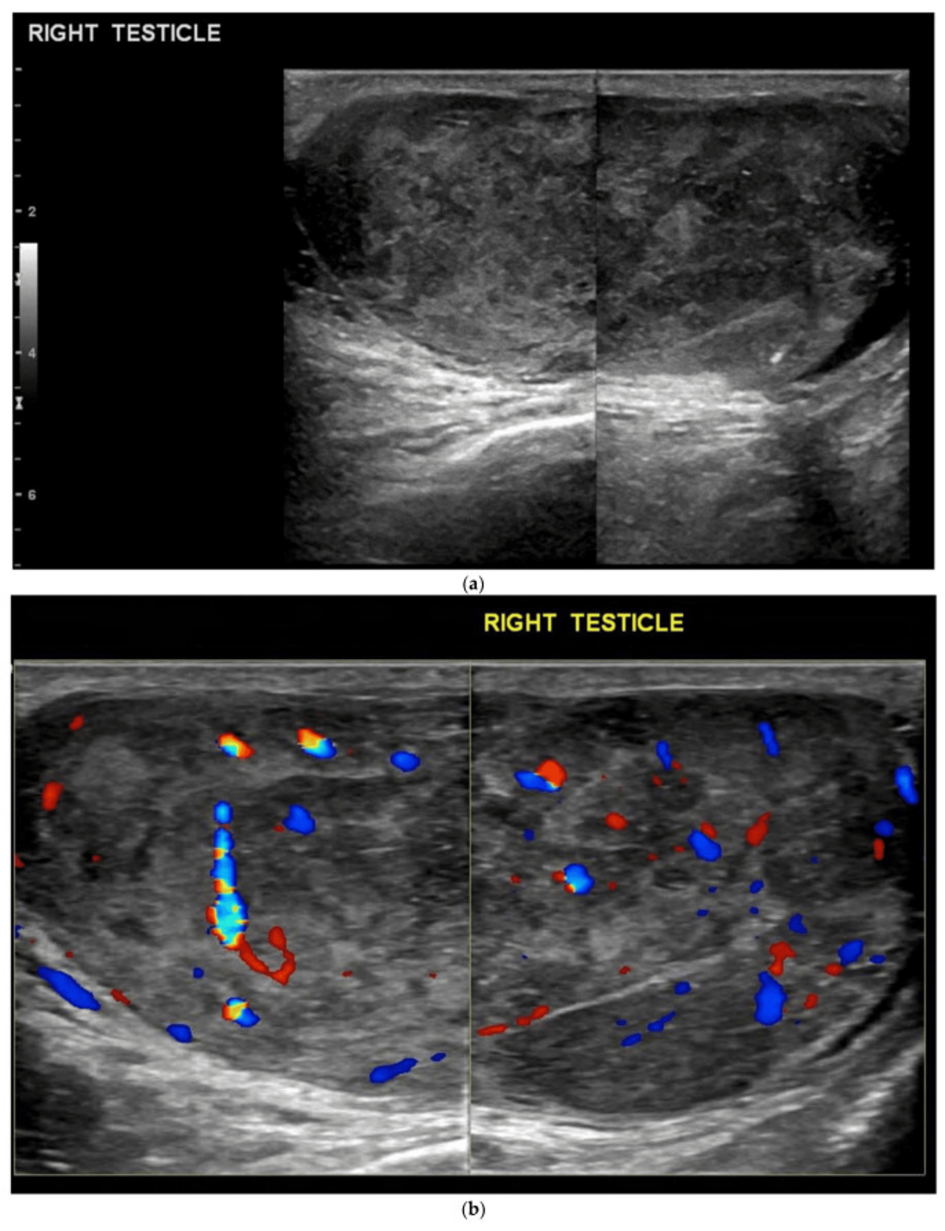
Illustrative ultrasound image of a heterogeneous intratesticular mass from a previously published open-access article This figure does not correspond to our patient but is provided to illustrate the typical sonographic appearance of a solid malignant testicular lesion (Cancers, MDPI, CC-BY license) [11]. (a) B-mode ultrasound shows a heterogeneous large mass infiltrating and enlarging the right testis, containing areas of calcification and small cystic foci.  (b) Color Doppler ultrasound demonstrates increased vascularity throughout the mass.

The ultrasound report clearly described a 41-mm heterogeneous intratesticular mass consistent with malignant infiltration, although the original images were not available for archiving. To illustrate the typical sonographic appearance of testicular metastasis, we have included an example image from a previously published open-access case report (Figure 2). This figure is used solely for educational comparison, as the ultrasound image from our patient was not available for archiving.

Several cases of testicular metastases from SCLC have been documented [3-12]. Early reports by Stein et al. [3] and Rosser & Gerrard [8] provided clear pathological confirmation. More recent cases include Dincer et al. [7], who described a 52-year-old patient with metastatic SCLC and testicular involvement confirmed by orchiectomy.

Most reported cases share common features such as elderly patients, testicular presentation during metastatic progression, poor overall prognosis and sanctuary-site behavior due to the blood-testis barrier.

To better characterize the clinical profile and outcomes of SCLC-related testicular metastases, we compiled a comparative table of all available published cases, shown in Table 2.

**Table 2:** Summary of previously published cases of testicular metastasis from lung carcinoma compared with the present case This table provides a comparative overview of all available published cases of testicular metastasis originating from lung carcinoma, including SCLC, squamous cell carcinoma, and adenocarcinoma. For each case, patient age, laterality, timing of testicular involvement, histologic confirmation, treatment approach, and clinical outcome are summarized. The present case is included for direct comparison, highlighting its unique features such as lack of histologic confirmation and management through systemic therapy rechallenge and palliative radiotherapy. SCC: Squamous cell carcinoma

Author/Year	Age	Laterality	Timing	Histologic Confirmation	Treatment	Outcome
Stein et al., 1989 [3]	63	Unilateral	Metachronous	Yes (orchiectomy)	Chemotherapy	Rapid deterioration
Rosser & Gerrard, 2000 [8]	69	Unilateral	Metachronous	Yes	Chemotherapy	Death
Dincer et al., 2021 [7]	52	Unilateral	Metachronous	Yes	Orchiectomy + chemotherapy	Progression
Buck et al., 2015 (lung SCC) [9]	69	Unilateral	Synchronous	Yes	Surgery + chemotherapy	Death
Uchida et al., 2003 (lung SCC) [10]	62	Unilateral	Metachronous	Yes	Orchiectomy	Death
Kaplan et al., 2012 (lung SCC) [12]	58	Unilateral	Synchronous	Yes	Surgery	Death
Ozeki et al., 2019 (lung adenocarcinoma) [13]	63	Bilateral	Metachronous	Yes	Nivolumab	Partial response
Present case (Alami et al.)	61	Right unilateral	Metachronous	No (clinical suspicion)	EP rechallenge + palliative RT	Stabilization

Several mechanisms have been proposed to explain testicular metastasis. These include arterial embolization (considered the most likely in SCLC) [4], retrograde venous or lymphatic spread, direct extension from adjacent organs, and dissemination through pre-existing cavities.

Testicular tissue may act as a “pharmacologic sanctuary site,” limiting chemotherapeutic penetration due to the blood-testis barrier [13], leading to isolated progression despite systemic control.

Clinicians should be aware of potential testicular involvement in patients with SCLC, especially older patients presenting with new scrotal symptoms. Isolated testicular progression may indicate sanctuary-site escape and justify both clinical examination and, when appropriate, scrotal ultrasound.

Systemic chemotherapy remains the mainstay of treatment. Our approach is consistent with the NCCN Clinical Practice Guidelines for Small Cell Lung Cancer, which recommend EP rechallenge for sensitive relapse occurring more than six months after completion of initial therapy [14].

Orchiectomy may be indicated in oligometastatic disease or for diagnosis, pain control, or debulking. This patient's treatment focused on systemic control and palliation due to extensive metastatic burden.

In patients with disseminated SCLC and rapidly progressive disease, invasive diagnostic procedures such as orchiectomy may offer limited therapeutic benefit and may be clinically inappropriate, a situation described in other published cases.

In summary, this case highlights several important learning points. Although testicular metastasis is uncommon, it should be considered in elderly patients with SCLC who develop new scrotal symptoms (swelling, testicular pain...). The testis may function as a pharmacologic sanctuary site due to the blood-testis barrier, potentially allowing isolated progression despite otherwise effective systemic therapy. When biopsy is not feasible, diagnosis must rely on the clinical context and ultrasound findings, while acknowledging their limitations. Finally, in cases of sensitive relapse occurring more than six months after initial treatment, EP rechallenge remains an evidence-supported therapeutic option.

This case has several limitations. A major limitation of this case is the absence of histologic confirmation. However, in patients with disseminated SCLC and rapidly progressive disease, invasive diagnostic procedures such as orchiectomy may offer limited clinical benefit and may be inappropriate, as reported in previous cases. Another limitation of this report is the absence of the actual ultrasound images, which could not be retrieved from our institutional archives. Nevertheless, the radiology report provided clear documentation of a solid intra-testicular mass, allowing clinical correlation with previously reported cases of testicular metastasis from SCLC. In addition, an illustrative open-access ultrasound image from the literature has been included to demonstrate the typical appearance of such lesions. These limitations should be considered when interpreting the diagnostic conclusions.

## Conclusions

Testicular metastasis from SCLC is rare and diagnostically challenging, particularly in the absence of histologic confirmation. In elderly patients with known metastatic SCLC who develop testicular enlargement, clinicians should consider testicular metastasis within the differential diagnosis. This case also highlights practical diagnostic challenges encountered in low-resource or emergency contexts, where complete imaging archiving may not be available. Despite the limitations related to the absence of biopsy and lack of retrievable ultrasound images, this case underscores the importance of clinical context, the sanctuary-site phenomenon, and careful physical and ultrasound evaluation in guiding diagnosis and management.
